# Incidence of subsequent primary cancers and radiation-induced subsequent primary cancers after low dose-rate brachytherapy monotherapy for prostate cancer in long-term follow-up

**DOI:** 10.1186/s12885-020-06960-9

**Published:** 2020-05-20

**Authors:** Kristiina Vuolukka, Päivi Auvinen, Jan-Erik Palmgren, Sirpa Aaltomaa, Vesa Kataja

**Affiliations:** 1 Cancer Center, Kuopio University Hospital, PO Box 100, FI-70029 Kuopio, Finland; 2 University of Eastern Finland, Kuopio, Finland; 3 Department of Urology, Kuopio University Hospital, PO Box 100, FI-70029 Kuopio, Finland; 4 Central Finland Health Care District, Central Finland Central Hospital, Adm Bldg 6/2, FI-40620 Jyväskylä, Finland

**Keywords:** Prostate cancer, Low dose-rate brachytherapy, Subsequent primary cancer, Radiation induced subsequent primary cancer

## Abstract

**Background:**

As aging is the most significant risk factor for cancer development, long-term prostate cancer (PCa) survivors have an evident risk of developing subsequent primary cancers (SPCs). Radiotherapy itself is an additional risk factor for cancer development and the SPCs appearing beyond 5 years after radiotherapy in the original treatment field can be considered as radiation-induced subsequent primary cancers (RISPCs).

**Methods:**

During the years 1999-2008, 241 patients with localized PCa who underwent low dose-rate brachytherapy (LDR-BT) with I125 and were followed-up in Kuopio University Hospital, were included in this study. In this study the incidences and types of SPCs and RISPCs with a very long follow-up time after LDR-BT were evaluated.

**Results:**

During the median follow-up time of 11.4 years, a total of 34 (14.1%) patients developed a metachronous SPC. The most abundant SPCs were lung and colorectal cancers, each diagnosed in six patients (16.7% out of all SPCs). The crude incidence rate of RISPC was 1.7% (*n* = 4). Half of the SPC cases (50%) were diagnosed during the latter half of the follow-up time as the risk to develop an SPC continued throughout the whole follow-up time with the actuarial 10-year SPC rate of 7.0%. The crude death rates due to metachronous out-of-field SPCs and RISPCs were 50 and 50%, respectively.

**Conclusion:**

The crude rate of SPC was in line with previously published data and the incidence of RISPC was very low. These results support the role of LDR-BT as a safe treatment option for patients with localized PCa.

## Background

With over 1.2 million new cases diagnosed worldwide annually, prostate cancer (PCa) is the second most common solid tumor in males [1]. As aging is the most significant risk factor for cancer development, long-term PCa survivors have, per se, an evident risk of developing multiple subsequent primary cancers (SPCs). Radiotherapy (RT) is also a risk factor for cancer development and SPCs appearing beyond 5 years after RT in the original treatment field can be considered as radiation-induced subsequent primary cancers (RISPCs) [2, 3]. There is also an increased risk of SPCs outside the radiation field either because of radiation scattering or from radiation-induced genetic alterations without direct in-field exposure. The risks of SPC and RISPC become even more relevant as the median overall survival (OS) of the RT treated patients with localized PCa now exceeds 12 years [4]. Thus, not only the efficacy and the adverse event profile of the treatment approach, but also the potential risk of SPC and RISPC may influence the selection of the treatment modality for PCa.

Several treatment options are available for localized PCa, yet no consensus regarding the optimum primary treatment exists [5, 6]. The RT modality may have an effect on the incidence of both SPCs and RISPCs as this is well supported with data from risk analyses of PCa patients treated with different RT modalities [3]. Most of the studies have included patients treated with external beam radiotherapy (EBRT) in varying planned target volumes, fractionation and techniques, while some have included patients treated with EBRT in combination with either low dose-rate brachytherapy (LDR-BT) or high dose-rate boosts. The extensive systematic reviews conducted by Murray et al. and Wallis et al. revealed a small increased risk of SPC in irradiated PCa patients with the risk of RISPC being estimated to be small but increasing with time [2, 3]. Since they have very heterogenic cohorts with varying follow-up times, these results are difficult to interpret and it is not possible to draw definitive conclusions regarding different RT modalities and the actual risks of SPCs and RISPCs.

Considering the irradiated volume, it could be expected that the SPC risk after LDR-BT would be lower than the corresponding risk after EBRT due to the lack of scattering radiation and the low risk of radiation leakage. The International Commission on Radiological Protection has made this conclusion already in 2005 [7]. Abdel-Wahab et al. have confirmed this finding, as significantly (*p* < 0.0001) fewer SPCs were diagnosed among patients treated with brachytherapy as compared to EBRT, 4.7, and 10.3% respectively, in the SEER tumor registry data from the years 1988–2002 [8].

Despite the increasing role of moderate and ultra-hypofractionated EBRT and stereotactic body radiotherapy (SBRT) techniques in the management of localised PCa, LDR-BT is still a valid treatment modality for low and favourable profile (Gleason score 3 + 4) intermediate-risk patients and is offered in several centers around the world. LDR-BT enables a single session treatment with very high total doses (145–150 Gy) to the target and steep dose gradients to the organs at risk surrounding the prostate. With technical advances in imaging and seed implantation, the LDR-BT in treating localized PCa has gained further popularity.

In Kuopio University Hospital (KUH), LDR-BT for patients with localized low and intermediate-risk PCa was initiated in 1999. The long-term efficacy and toxicity results of LDR-BT in this patient cohort has been published recently [9]. The aim of this study was to analyze the overall SPC rate and the incidence of potential RISPCs among Nordic patients with localized PCa with an 8–16 years follow-up time.

## Methods

During the years 1999–2008, 241 patients from KUH district with localized PCa were treated with LDR-BT monotherapy with I^125^ seeds according to ESTRO/EAU/EORTC guidelines [10]. All relevant patient data was scrutinized to identify subsequent cancer diagnoses (excluding basal cell carcinoma) evolving after LDR-BT. An SPC was defined as a metachronous primary cancer developing a minimum of 6 months after LDR-BT and an RISPC was defined as an in-field SPC developing a minimum of 5 years after LDR-BT and both SPC and RISPC representing a different histological type to the original cancer [2, 3]. For the purposes of this study, the SPCs were categorised as in-field (IF) malignancies including all SPCs arising within the true pelvis (i.e. in the prostate, the bladder, the rectum or the anus) and as out-of-field (OOF) cancers including all extra pelvic SPCs. The time to the SPC and RISPC was defined as the time from the date of LDR-BT to the date of the clinico-pathological diagnosis of SPC and RISPC, respectively. The statistical analyses were performed with SPSS Statistics 22 software (SPSS, Chicago, IL).

## Results

The patient demographics and clinical data on the PCas are shown in Table 1. During the follow-up time (median follow-up (mFU) of 11.4 years, range 8–16 years), a total of 34 patients developed a metachronous subsequent primary cancer, the crude incidence rate of SPC thus being 14.1%. Two of the patients developed also another subsequent cancer. The most abundant SPCs were lung and colorectal cancers both diagnosed in six patients and each representing 16.7% of all the SPCs (Table 2). The crude incidence rate of RISPC was 1.7% as four SPCs fulfilled the criteria for RISPC (Tables 2 and 3).

**Table 1:** Patient demographics

	n (%)
All patients	241 (100)
**Age (y)**	
Median	65
Range	46–79
**T classification at diagnosis**	
T1	148 (61.4)
T2a	75 (31.1)
T2b	9 (3.7)
T2c-3	9 (3.7)
**Gleason score at diagnosis**	
≤ 6	208 (86.3)
7	26 (10.8)
≥ 8	2 (0.8)
NA	5 (2.1)
**PSA at diagnosis (ng/ml)**	
PSA ≤ 10	165 (68.5)
PSA 10.1–19.9	71 (29.5)
PSA ≥ 20	5 (2.1)
**D’Amico risk group**	
Low	142 (58.9)
Intermediate	85 (35.3)
High	14 (5.8)
**Short term ADT**	
No	188 (78.0)
Yes	53 (22.0)

*PSA* Prostate-specific antigen, *ADT* Androgen deprivation therapy, *NA* Not available

**Table 2:** Metachronous SPCs, time to SPCs and locations of the SPCs after LDR-BT

SPCs, organs involved	n	%	< 5 years	≥ 5 years	OOF	IF
All	36	100	13	23	32	4
Lung	6	16.7	2	4	6	
Colon	5	13.9	2	3	5	
Skin^a^	5	13.9	3	2	5	
Hematologic	4	11.1	3	1	4	
Bladder	2	5.6		2		2
Rectum	1	2.8		1		1
Prostate (SCC)	1	2.8		1		1
Other^b^	12	33.3	3	9	12	

*SPC* Subsequent primary cancer, *LDR-BT* Low dose-rate brachytherapy, *SCC* Squamous cell cancer, *OOF* Out-of-field, *IF* In-field

^a^Fibrosarcoma, Squamous cell cancer, Melanoma

^b^Head and neck (*n* = 3), Liver (*n* = 2) and one of each: Brain, Eye melanoma, Esophagus, Pancreas, Kidney, Malignant schwannoma (muscle), Sarcoma (hand)

**Table 3:** Subsequent primary cancers fulfilling the criteria of an radiation-induced subsequent primary cancer

Age at the time of LDR-BT (y)	In-field SPCs diagnosed ≥ 5 years after LDR-BT	Time to an in-field SPC (y) after LDR-BT	Status at the cut-off date	TTD from the diagnosis of an RISPC due to an RISPC (mo)
60	squamous cell carcinoma of the prostate, grade 2	9.3	death due to the RISPC	7
74	invasive urothelial cancer of the bladder, grade 3, pT1N0M0	9.8	death due to the RISPC	5
57	adenocarcinoma of the rectum, grade 2, pT2N0M0	9.9	alive	
72	papillary urothelial neoplasia of the bladder	13.3	alive	

*LDR-BT* Low dose-rate brachytherapy, *SPC* Subsequent primary cancer, *RISPC* Radiation induced subsequent primary cancer, *TTD* Time to death, *y* years, *mo* months

**Fig. 1:**
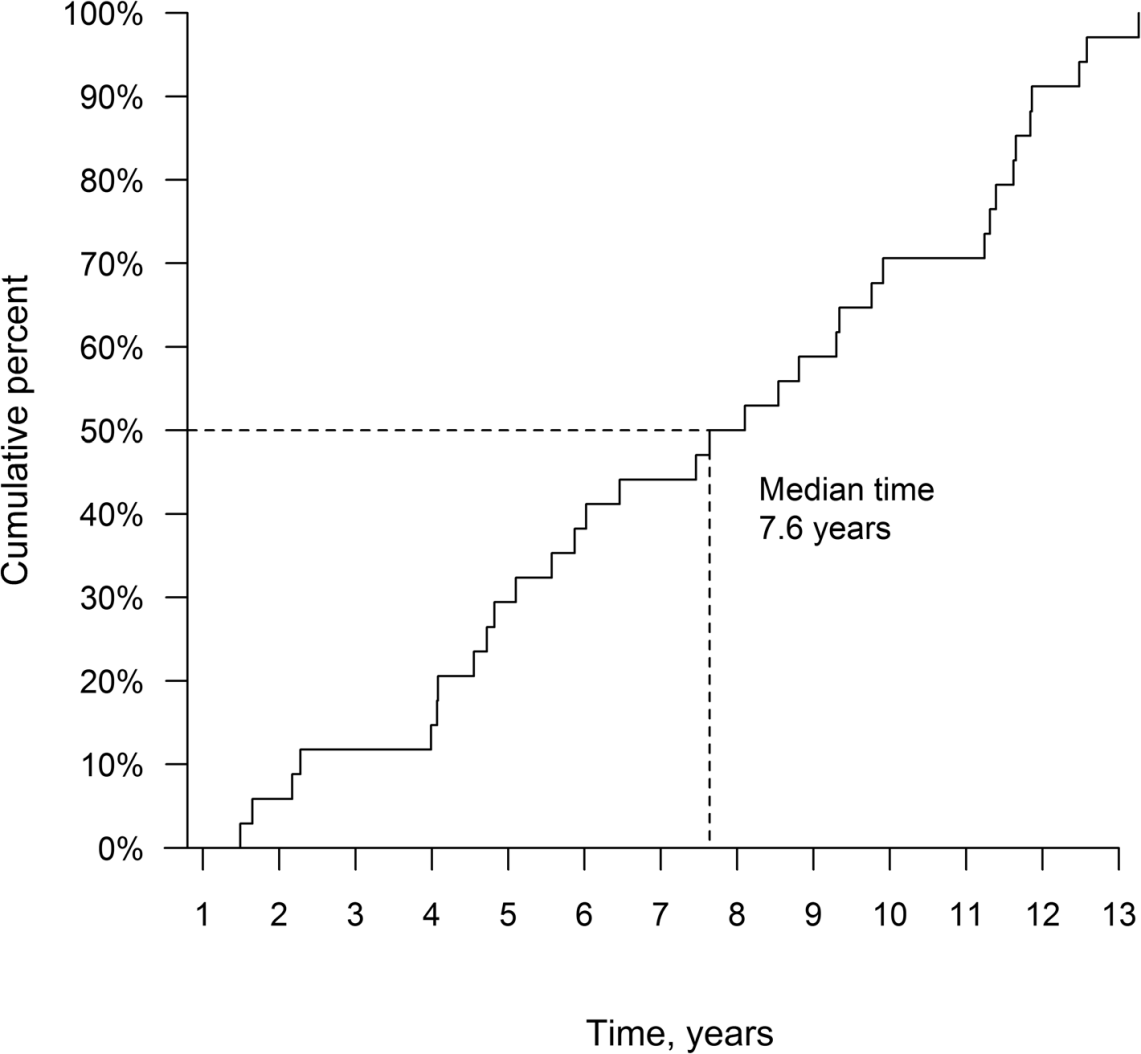
The incidence of second primary cancers (SPCs) during the follow-up

As shown in Fig. 1, every second (17 cases, 50%) of the SPCs was diagnosed during the first 7.6 years of the follow-up and the risk to develop an SPC continued throughout the follow-up time. The actuarial 10-year SPC rate was 7.0%. Twenty-three of the SPCs developed more than 5 years after LDR-BT, but only four of these developed in the IF area (Table 2). The median times to develop a metachronous OOF SPC and an RISPC were 7.0 years (range, 1.5–12.6 years) and 9.8 years (range, 9.3–13.3 years) after LDR-BT, respectively. The third primary cancers, a squamous cell carcinoma of the vocal cords and an adenocarcinoma of the hepatic flexure of the colon, were diagnosed at 12.6 and 12.8 years after LDR-BT, respectively.

At the mFU of 11.4 years, the OS rates for all patients (*n* = 241), patients with no metachronous SPC (*n* = 207), patients with a metachronous OOF SPC (*n* = 30) and patients with an RISPC (*n* = 4) were 66.4, 69.1, 50 and 50%, respectively. Out of the 81 deaths during the follow-up, 53 (65.4%) were due to co-morbidities or trauma and 11 (13.6%) due to PCa. Seventeen (21.0%) patients died due to a malignancy other than PCa: 15 due to a metachronous OOF SPC and two due to an RISPC. The actuarial 10-year death-rates due to PCa, metachronous OOF SPC and RISPC were 3.0, 3.0% and < 1.0%, respectively.

## Discussion

In this real-life cohort of 241 PCa patients with a median age of 65 and an mFU of 11.4 years after LDR-BT monotherapy, 34 (14.1%) patients were diagnosed with an SPC. The incidence is low, but clinically meaningful, since every second patient diagnosed with an SPC died from this malignancy. The actuarial 10-year death-rate due to an SPC was equal to the actuarial 10-year death-rate due to PCa itself. The majority of the SPCs were diagnosed during the latter half of the follow-up period emphasizing the significance of a long follow-up time.

In European studies, the values of 10-year cumulative incidence of any SPC are concordant with our results [11, 12]. A study from Australia, a country with a relatively higher incidence of melanoma, reported a 10-year cumulative SPC incidence of 18.5% [13]. However, with the exception of the study by Cosset et al. from France, little is known about the SPC incidence in cohorts with mFU times extending over 10 years after LDR-BT monotherapy [14]. With 675 patients treated with LDR-BT and the mFU of 11.0 years, the 10-year cumulative incidence of secondary malignancies was 7%, similar to our results [14]. Altogether, several studies with large patient cohorts and varying follow-up times have concluded there is no excess risk of SPC among patients treated with LDR-BT due to PCa [11, 12, 14, 15].

As RT itself is a risk factor for cancer development, the risk of RISPCs in the pelvic area is considered as one of the possible late adverse events after curative intended LDR-BT. Compared to the general population, an increased risk of bladder cancer after LDR-BT has been reported in single-centre cohorts from the Netherlands, the UK and Australia, but only during the first four to 5 years after LDR-BT and among patients under 60 years of age [11–13]. This might be due to increased urological surveillance after the treatment [11, 13], as the incidences of bladder cancer were higher than expected also among RP patients, the lead-time or screening bias may explain these results [12]. However, the influence of radiotherapy as a possible risk factor for a second primary cancer cannot be excluded.

In European single-centre LDR-BT-cohorts, no increased risk of rectal cancer has been observed [11, 12, 14]. Hamilton et al. did not find any increased risk of pelvic SPCs among PCa patients treated with BT as compared to those treated with RP [15] and Cosset et al. confirmed a very low risk, if any, of RISPC after LDR-BT [14]. In our cohort, the cumulative incidence of RISPC was very low, since only four patients developed an RISPC. The actuarial 10-year death-rate due to RISPC was < 1%. In SEER based analysis by Abdel-Wahab, there were no statistically significant differences found in the incidence of RISPCs between different RT-groups (EBRT, BT or their combination) showing that LDR-BT is as safe as other RT treatments [8].

In addition to aging and effects related to the treatment, radiation in this context, SPCs can arise because of a genetic predisposition and a shared etiological background. Furthermore, lifestyle factors, i.e. smoking habits, and workplace exposure to carcinogenic chemicals may contribute to the SPC incidence, also in organs at risk. Hence, a new SPC does not necessarily have a causal relation to the previous LDR-BT. The only undisputable RISPC after LDR-BT in our cohort is the grade 2 SCC of the prostate, which has previously been reported as a case report [16]. Patients may also have synchronous malignancies and sometimes it is difficult to determine which is the primary and which is the subsequent primary cancer. In our cohort, six (2.5%) patients had a medical history of previous cancer, which categorizes the PCa itself to the position of a subsequent primary cancer.

As the nature of the low-risk and favorable-profile intermediate-risk PCa is non-aggressive and the efficacy of LDR-BT for this particular malignancy is shown to be excellent [17–19], patients do survive and live long enough for some of them to develop another malignancy. SPCs seem to be deadly diseases, irrespective of the location as in a cohort of 2418 BT-treated men with mFU of 5.8 years, 41% of the patients developing an SPC died due it [15]. In our study with mFU almost twice as long, of the patients diagnosed with an OOF SPC, 50% (15 patients out of the 30 patients with an OOF SPC) died due it. As the absolute numbers of SPCs are low, the clinical burden is not overwhelming. On the other hand, at 10 years after LDR-BT, the risk of dying due to a metachronous SPC was equal with the risk of dying due to the primary cancer itself. Equally, with RISPCs, in our cohort the numbers are very small, but nonetheless the 50% death-rate due to the RISPCs is noteworthy.

In our cohort, every second metachronous SPC was diagnosed during the first 7.6 years. The risk to develop an SPC continued steadily, if not even more steeply, over the follow-up period, as shown in Fig. 1, and the same phenomenon has been reported previously in studies with European and Western patient populations [20, 21]. As the OS of PCa patients is lengthening and the aging is a major risk factor for cancer development in general, this observation is not unexpected. At the cut-off point, the majority of the patients (160 out of 241, 66.4%) in our cohort were still alive and could possibly develop an SPC or an RISPC in the future. The authors are aware of the main limitations of the study: the study is retrospective and the patient cohort is limited in size. However, since the mFU exceeds 10 years and only a very long-term follow-up after RT will reveal the life-long risks of SPCs and RISPCs among the curatively treated cancer patients, we find our data valuable and these results worthy of reporting.

## Conclusion

With a median follow-up of 11.4 years in our real-life PCa patient cohort treated with LDR-BT, the follow-up time is sufficient to report the incidences of SPCs and possible RISPCs. The crude rate of SPC was in line with previous data and of the same order of magnitude as the cancer incidence among a similar aged general Finnish male population [22]. In addition, the number of RISPCs was very low as only four patients out of 241 developed an in-field SPC. In conclusion, our results with very long follow-up support the safety of LDR-BT as a treatment option for patients with localized PCa.

## Data Availability

The data that support the findings of this study are available from the patient records of the Kuopio University Hospital, but restrictions apply to the availability of these data, which was used under license for the purposes of the current study only, and so are not publicly available. The data in anonymized form is available from the authors upon reasonable request and with permission of Kuopio University Hospital.

## Abbreviations

EBRT: External beam radiotherapy
IF: In-field
KUH: Kuopio University Hospital
LDR-BT: Low dose-rate brachytherapy
mFU: Median follow-up
OOF: Out-of-field
OS: Overall survival
PCa: Prostate cancer
RISPC: Radiation-induced subsequent primary cancer
RT: Radiotherapy
SBRT: Stereotactic body radiotherapy
SCC: Squamous cell cancer
SPC: Subsequent primary cancer
